# Stillbirth in Lao PDR: a healthcare provider perspective

**DOI:** 10.1080/16549716.2020.1786975

**Published:** 2020-08-03

**Authors:** Molina Choummanivong, Sediqa Karimi, Joanne Durham, Vanphanom Sychareun, Vicki Flenady, Dell Horey, Fran Boyle

**Affiliations:** a Faculty of Public Health Department, University of Health Sciences, Vientiane, Lao People’s Democratic Republic; b School of Public Health, The University of Queensland, Brisbane, Australia; c Centre of Research Excellence in Stillbirth, Mater Research Institute, The University of Queensland, Brisbane, Australia; d School of Public Health and Social Work, Queensland University of Technology, Brisbane, Australia; e Public Health, School of Psychology and Public Health, La Trobe University, Melbourne, Australia; f Institute for Social Science Research, The University of Queensland, Brisbane, Australia

**Keywords:** LEARN: Sexual Reproductive Health, ANC and Nutrition, Bereavement care, Lao PDR, low- and middle-income countries, stillbirth, healthcare professionals, grief

## Abstract

**Background:**

Stillbirth is a major global concern. However, most research has been conducted in high-income countries. Understanding of the experience and management of stillbirth in low-middle income countries is needed.

**Objective:**

This qualitative study explored health professionals’ experiences of providing stillbirth care in the Lao People’s Democratic Republic, a lower-middle-income country in South-East Asia.

**Methods:**

In-depth interviews were conducted with 33 health professionals (doctors, midwives and nurses) and thematic analysis was undertaken.

**Results:**

All participants acknowledged stillbirth as a concern, but its incidence and causes were largely undocumented and unknown. A lack of training in managing stillbirth left health professionals often ill-equipped to support mothers and provide responsive care. Social stigma surrounds stillbirth, meaning mothers found limited support or opportunities to openly express their grief.

**Conclusions:**

Better awareness of stillbirth causes could promote more positive experiences for healthcare providers and parents and more responsive healthcare. This requires improved training for healthcare professionals and awareness raising in the wider community.

## Background

The Sustainable Development Goals (SDGs) are likely to be important drivers of the direction and level of resource commitment to health at the global and national level [1]. The estimated global stillbirth rate (≥28 completed weeks’ gestation) is 18.4 per 1000 total livebirths or approximately 2.6 million deaths annually [1]. Many stillbirths, however, are preventable with high-quality care during pregnancy and childbirth [2,3]. Inequalities exist, however, within and between countries in access to high-quality maternal care, with most stillbirths occurring in lower- or lower-middle-income countries (LMICs) where many women do not have access to quality healthcare [2,4–6].

Despite LMICs carrying the largest stillbirth burden, research into the experience of stillbirth in LMICs is sparse. In their review of studies of experiences of stillbirth in LMICs, Shakespeare, Merriel [7] found only four studies focussing on healthcare professionals. Available evidence suggests major shortcomings in the bereavement care provided due to a lack of health system resources and adequately trained healthcare providers [7]. Similarly, a review of seven qualitative studies of women’s lived experiences of stillbirth in Asia-Pacific countries concluded that health systems and health professionals are ‘often at a loss’ as to the most appropriate care [8].

The Lao People’s Democratic Republic (Lao PDR), is an LMIC within South-East Asia. Maternal and child health is determined a priority by the government and the country has made impressive gains over the last decade. These gains are attributed to increased family planning, facility-based delivery, improved breastfeeding practices, and general socio-economic uplift [9]. The MMR at estimated 197 deaths/100,000 live births, however, remains the highest in the region [9]. The estimated stillbirth rate in Lao PDR is 23.7 per 1000 births [10] which is high when compared with higher-income countries (HICs) such as Australia (3 per 1000 live births) and the global target of 12 or fewer per 1000 live births by the year 2035. Despite the attention to maternal and neonatal health, stillbirths are unacknowledged. The purpose of this study was to provide insights into healthcare professionals’ knowledge and management of stillbirth in Laos PDR. Developing appropriate interventions to reduce stillbirth and to support healthcare professionals, mothers and their families requires an understanding of how stillbirth is managed in healthcare facilities [7]. Before proceeding, however, we provide a brief overview of the healthcare system.

## The Laotian healthcare system

The healthcare system in the Lao PDR operates at three administrative levels: central (Ministry of Health, MOH); provincial (provincial health offices, PHOs); and district level (district health offices, DHOs). The healthcare system is predominately public, although there some private providers in larger urban areas and a large number of private pharmacies and clinics. Tertiary and specialised hospitals are based in Vientiane Capital with second-tier hospitals located in each provincial capital, and the first referral level hospital at the district level. At the sub-district level, health centres serve multiple surrounding villages [11,12]. In addition, the military and police sectors provide healthcare services for their own cadres, their family and parts of the local community. Healthcare facilities and equipment are often old and poorly maintained. Across the healthcare sector, there is a severe shortage of skilled health workers (doctors, nurses, midwives, lab technicians) [11]. The most highly qualified health personnel and specialists work at central- and provincial-level health facilities [11]. There is, however, a deficit of specialist medical doctors such as obstetricians and medical doctors with no specialist training may work in specialist areas with expertise developed through experience. Also, as elsewhere, public sector staff may also work in the private sector [11,12].

To improve maternal and neonatal health, the government, under its National Insurance Scheme, provides pregnant women free access to maternal health services including treatment costs, transportation (with a co-payment) and an incentive to attend four antenatal care (ANC) appointments [13]. The government also invested in midwifery training, resulting in the number of births with a skilled birth attendant increasing from 37.5% in 2011 to 32% in 2012 to 53% in 2017 [13]. As with most of the other cadres within the healthcare system, however, there remains a critical shortage of midwives and many healthcare centres, especially in rural and remote areas where many women continue to deliver without a skilled birth attendant [9,11,14].

## Methods

This study was conducted in Vientiane Province in the northwest of the country. The Province contains 10 districts and Vientiane Capital City. The province is a mix of urban, peri-urban and rural areas. This study involved in-depth, semi-structured, face-to-face interviews with healthcare providers. Interviews were conducted in English or Lao, depending on the participant’s preferred language. Interviews were led by SK, who worked with an interpreter and co-researcher, MC, from the University of Health Sciences in Vientiane who ensured the cultural and linguistic appropriateness of the questions and assisted in interpreting as needed. Interviews took place in June 2018.

The first author (MC) is a Laotian and an experienced qualitative researcher with a background in medicine and public health. Her background enabled MC and SK to quickly develop rapport and trust which is likely to have helped participants to provide more accurate and honest data [15]. SK is a registered nurse with post-graduate training in qualitative public health research. While now living and working in Australia, SK was born in a rural district in Afghanistan, an LMIC with poor maternal and child health outcomes and limited access to healthcare. Reflexively in the research process was operationalised via observation, reflection and through discussions with the other co-authors and colleagues [16]. MC’s medical background and clinical experience and discussion between MC and SK immediately after each interview enhanced data integrity. SK also maintained a journal throughout, allowing her to examine her own learnings, values and feelings and facilitate a deeper engagement with participants’ experiences [16,17].

The interview questions drew on work previously conducted for The Lancet Ending Preventable Stillbirths series, including a large multi-country online survey of health professionals that centred on global priorities for stillbirth research [18]. As an exploratory study, we used a semi-structured interview guide consisting of a series of broad open-ended questions designed to elicit the views and experiences of participants and identify issues they felt were most relevant. The broad topics related to participants’ overall perspective and understanding of stillbirth, how stillbirth is diagnosed, bereavement care, stillbirth management and impact on healthcare workers and participants’ suggestions and recommendations (Supplementary Material 1). As well as the interviews contained in the topic guide, participants were invited to discuss issues relevant to them within the scope of the overall research topic. Interviews were digitally recorded and transcribed verbatim in English. Where interviews were translated from Lao to English, as well as checking for accuracy, transcripts were also checked against MC and SK’s notes. Where there were discrepancies, they were rechecked against the recordings.

Non-probabilistic purposive sampling was used to identify healthcare facilities and participants. The sample was determined based on research objectives and where, based on experience and subjective knowledge of the facilities, participants would be motivated to participate in the research and provide rich detail. At the central level, the three main hospitals specialising in women’s and mothers’ health were selected. Hospitals at the provincial level (n = 1) and district level (n = 5) were also included for a range of perspectives. Purposive sampling was used to select healthcare professionals directly or indirectly involved in the care of a mother who had experienced stillbirth. Given many healthcare facilities do not have specialist staff, non-maternity health workers, including medical doctors and nurses, were also selected. All interviews were conducted at the interviewee’s healthcare facility in a place where privacy could be assured and ranged in length from 25 to 90 minutes.

Thematic analysis was applied to the interview transcripts following the approach outlined by Terry, Hayfield [19]. This iterative process allows for a combination of inductive and deductive techniques and involves five steps: *familiarisation with the data*, which involves reading and re-reading the interview transcripts; *generating codes* as patterns and ideas were identified; *constructing themes* to summarise groups of codes in meaningful ways and build a narrative from the data; *reviewing potential themes*; and finally *defining and naming* the final set of themes. SK led the analysis, supported by regular discussion with other members of the research team throughout all phases.

Ethics approval was obtained from the Human Research Ethics Committee at the University of Queensland (#2017000978) in accordance with the Australian National Statement on Ethical Conduct in Human Research. Local ethics approval was obtained from the Lao PDR University of Health Sciences Ethical Review Board following their review of translated copies of the research proposal executive summary, consent form and interview guidelines. Written informed consent was obtained from all study participants.

## Results

All participants who were invited to participate agreed to do so and in total, 33 interviews were conducted with 11 doctors, 7 doctor/obstetricians, 6 midwives, 5 nurse/midwives and 4 nurses. All participants had several years of clinical experience, ranging from 6 to 30 years: 11 had at least 10 years, and 17 had 20 years or more. For non-specialists, while maternity care was part of their work, as generalists, their years of experiences were not exclusively in maternity care. Two-thirds of those interviewed [20] were women. Some participants working at the central level also had experience at the provincial and district levels.

Three main themes were identified: stillbirths not counted, reviewed or audited; little support is available to mothers or families following stillbirth; and stillbirth challenges the emotional and professional wellbeing of healthcare providers. The results are organised according to these themes and illustrated using quotes from participants with the participant’s unique study identification number, discipline, and healthcare setting following each quote.

### Stillbirths are not counted, reviewed or audited

Study participants recognised stillbirth as a matter of concern, but substantial knowledge gaps existed about its prevalence and causes. Most participants reported they were only aware of stillbirth cases they had witnessed. As such, estimating the frequency of stillbirth was difficult.

There is no record of how many stillbirths … in the last year I experienced only six cases of stillbirth. These are the cases I was directly involved in. Other times when I am not in the hospital, it is not known. And cases outside the hospital are unknown. Home deliveries are not accounted for. (ID01, doctor, central hospital)

Stillbirths were likely to be under-reported as record-keeping was generally poor and annual audits were not conducted.

… there are no records kept at the national level and the number of stillbirths is never officially documented. Hence, the estimated number of stillbirths may only include those that happen in a healthcare facility, not the whole nation. So many stillbirths happen outside a healthcare facility and remain uncounted. So, no one ever reviewed the hospitals to find out how many stillbirths happened. (ID14, doctor, district hospital)

All respondents acknowledged that stillbirth reviews or formal investigations into their causes either nationally or at facility level were not routine and occurred only rarely.

… stillbirth review and investigation are based on the mother’s medical history from the antenatal clinic, if they have attended any. But this is only if the family requires information about how stillbirth [occurred]. Otherwise, there is no official review done here. (ID 25, doctor/obstetrician, district and central hospital).

We do not have a stillbirth review team, as we are a district hospital, no staff, no equipment, and no patients asked us to do, and no laws are allowed us to do the stillbirth review and investigation. (ID27, doctor, district hospital)

All respondents commented on shortcomings of the health system in Lao PDR and the implications both for preventing stillbirth and managing stillbirth when a death occurred. A lack of appropriate equipment in antenatal care clinics and obstetric departments was seen as increasing the risk of pregnancy-related complications, including stillbirth.

We do not have an operating theatre in this hospital, we only have sonokit machines with which we can monitor fetal heart rate. When mothers present, if we detect reduced fetal movement, there is not much we can do here, we send them to central hospital. If we had the equipment, we could monitor the mother and baby here and perform c-section and save the baby in time. But we cannot. And stillbirths keep happening. (ID05, midwife, district hospital)

In cases of stillbirth, confirming foetal death could be challenging as high-technology monitoring devices and senior health professionals were not readily available.

Sometimes it is very difficult for us to diagnose because we don’t always have an obstetrician or a midwife in our hospital all the time. So we rely on our monitoring of the fetal heart beat with our basic SonoKit and ultra sound machine. We ask the mother about her last menstruation cycle, we ask her if she had felt the baby was not moving, or if there was reduced fetal movement. (ID03, nurse, district hospital)

Autopsy services were generally non-existent across the country. Most interviewees believed that even if services were available, uptake would be low due to cultural taboos associated with autopsy. Many participants recognised the value of autopsy in establishing stillbirth cause but also understood the strong cultural barriers that existed.

… I think we have to go a long way before our people can understand the importance and the reason behind autopsy. To the people autopsy will mean, cut open the body of the dead and take everything out, which is a sin according to the gods. (ID12, doctor (obstetrician), central hospital)

We cannot investigate the cause of stillbirth. We will never know the cause. We cannot operate on the dead body. First because we do not have the right equipment and the right person skilled. Second, in my idea, and experience, the family and relatives will not allow to operate the dead baby because they believe that their baby will have the scar when born in the next life. (ID31, midwife, district hospital)

The healthcare professionals agreed there are no existing guidelines for stillbirth management nor prevention in the Lao context, and no specific or routine practices related to stillbirth care were identified.

### Little support is available to mothers and families following stillbirth

Healthcare providers explained that every death, including stillbirth, is accompanied by sorrow and grief. In their experience, mothers were usually experienced emptiness, loss, and sadness following stillbirth:

… from a recent case of stillbirth, the mother and the family were aware that the baby had died in the womb, they were numb and neutral. The mother had no choice, she did not speak and did not cry. It was as if, she was numb, and emotionless. (ID10, nurse/midwife, district hospital)

Most respondents observed mothers experienced a strong sense of guilt following a stillbirth.

… this mother was devastated for her loss and felt guilty for not being able to save her baby in her womb. She cursed herself and her motherhood. (ID19, midwife, central hospital)

There is no direct translation or interpretation of ‘bereavement’ in Lao, which sometimes makes it difficult for the healthcare professionals to describe their responses to ‘bereavement care’. However, all responses revealed that mothers are distressed by stillbirth. Sensitive and emotional support was needed but often not forthcoming due to health system limitations and wider sociocultural beliefs and practices.

Mothers are sad when stillbirth happens. We try to listen to their concerns and provide emotional support, but their stay with us, post-stillbirth is very short, only one day or two. After discharge, only very few mothers attend our clinics for ongoing support. Some mothers will have to suppress their sadness because of family and culture. Therefore, these mothers don’t always get the right emotional support. This make them vulnerable in the next pregnancies. (ID19, midwife, central hospital)

After a stillbirth, as the senior doctor I am expected to explain to the mother and family what happened. I understand that mothers need to be supported from the time the baby was suspected to be dead, but the majority of the time the mother does not receive that right care. In Lao, we have no bereavement care. So not all our staff are familiar with the right care. (ID07, doctor/obstetrician, provincial hospital)

In Lao PDR, it seemed to be uncommon for mothers or other family members to interact with their stillborn baby and mothers are usually not allowed to hold or even see their stillborn baby. Participants reported that many mothers plead for their husbands and in-laws to let them hold their baby, but often in vain. In some cases, husbands who may also be grieving, may transfer their disbelief and anger to the mother, potentially preventing her from engaging grieving, as in the example below:

Last year, I was involved in one stillbirth case, I went to tell the family and mother about stillbirth. The family was disappointed even though they knew that the baby was dead before they came to hospital, but they were angry. The mother cried and asked if she can see the baby. The husband interrupted and said, ‘you could not give birth to a live baby, what are you going to see a dead body for?’ (ID16, nurse, district hospital)

Most respondents felt that women faced stigma and blame following stillbirth. Negative social perceptions about stillbirth, lack of support from families, blaming the mothers and associations with superstitious causes were reported to make mothers of stillborn babies feel devalued, isolated and stigmatised.

… if she gives birth to a stillborn, she has to stay away from the family until she is clean from the evil spirit. She has to stay in a hut, away from the family home as the stillborn spirit will bring bad luck to the home. No one will talk to her, not even the husband. You can imagine how the mother feels – very alone and not supported. ID19, midwife, central hospital.

… But a lot of the time, a father will blame the mother for not keeping his child. So a mother feels incomplete and empty. (ID15, nurse/midwife, district hospital)

Many respondents believed that the severity of emotional distress depended on whether the baby was the first child or male, as these babies are more highly valued, particularly among the poor and in rural communities where boys become family breadwinners. Loss of a firstborn male child creates double devastation, especially for poor families.

The impact of stillbirth on the family when the child dies in the womb depends if it’s the first pregnancy or not. And also, if it is a boy. Then the stillbirth had very bad impact on the mother’s health and the economy of the family. When mother becomes pregnant many families look forward to baby boy, if he dies. They are disappointed. (ID32, doctor, district hospital)

Study participants also commented on the more direct economic impacts of stillbirth, particularly for low-income families living in rural areas because of the cost of travel to central and provincial hospitals. Families may also be required to pay a hospital if the body is left there.

There was one stillbirth case, the family refused to take the dead baby home due cultural beliefs. Therefore, they had to pay about 300,000 to 500,000 kip to the hospital to organise for the funeral (or to burn the cadaver). (ID27, doctor, district hospital)

### Stillbirth challenges the emotional and professional wellbeing of healthcare providers

The simultaneous occurrence of life and death coupled with the emotional distress of parents and families often left healthcare professionals involved in stillbirth care deeply affected. Strong emotions that negatively affected their personal and professional wellbeing, including frustration, guilt, self-blame and sadness, were frequently described.

As medical doctor we deal with life and death every day. But the death of a baby is different. It is mainly because it is difficult to witness the loss and sadness of the mother. Sometimes it is heart breaking. (ID18, doctor, central hospital)

These feelings may be magnified when a baby dies after the onset of labour, where the healthcare professional was directly involved in providing care at the time of death. This is illustrated by the following quote from a doctor when working at the district level where surgical capacity (for example, anaesthetics) is often lacking.

When I examined the mother and fetus, I could hear fetal heartbeat, but it was very weak and slow. We induced the mother into labour, she was pushing, but by the time the baby was delivered it was too late, the baby was dead. It was a stillbirth. I was frustrated with the system. Why was I frustrated with our poor health system? If we had the capacity to perform caesarean section, the baby could possibly have survived. I felt guilty and blamed myself, because the baby had a beating heart when the mother presented. This will haunt me. I was not able to save this human. (ID25, doctor/obstetrician, district/central hospital)

Many of the healthcare professionals, especially those with higher qualifications and expertise, often felt they were expected to solve every problem. Some reported that they felt pressured and their clinical abilities questioned in the event of a stillbirth.

I felt incompetent. If I had advanced training, maybe I could perform caesarean sections. (ID13, doctor, district hospital)

When families asked why their baby had died, they felt professionally insecure and unconfident, particularly if the cause was unclear. Fear of litigation was also experienced:

We are afraid that their relative will sue us. Specially, if I am the senior worker when the baby died, they ask me for answers. If the fetus died before thy presented to our health facility, we have no reason to be scared but if baby dies during labour then we have to be careful. (ID29, doctor, central hospital)

One midwife explained that midwives, being present at deliveries, are at particular risk of blame.

… if baby dies during delivery, then there is fear of litigation, staff would fear from being blamed by the family for the stillbirth, this will impact on their mental wellbeing and sometime will affect their ability to provide care. (ID06, midwife, district hospital)

Interviewees strongly voiced the lack of training for stillbirth management and care:

During my internship, I had my first case of stillbirth. I was not trained how to deal with stillbirth, I was shocked, and I was scared. I did not know how to communicate the news to the mother and family. (ID04, doctor, provincial hospital)

We want to help, but a lot of the time, we get frustrated because we do not know how to. (ID10, nurse/midwife, district hospital)

We have no specific training or education about stillbirth, therefore when it happens, we are unprepared. (ID14, doctor, district hospital)

## Discussion

To our knowledge, this is the first study to explore healthcare professionals’ experiences of providing care after stillbirth in Lao PDR. Our findings indicate that despite best efforts, healthcare providers face challenges in providing quality care around the time of stillbirth. Like many LMICs, the Lao PDR healthcare system appears to be limited in its current capacity and capability to provide responsive care following stillbirth. Our key findings, especially in relation to limited data and review, lack of bereavement care for mothers and inadequate training and emotional support for healthcare providers, coincide with themes common to the small number of studies focused on care after stillbirth in LMICs [6–8].

In this study as reported in other LMICs, participants reported poor record-keeping and an absence of an annual audit [4,6,7]. A review of data in the Mother and Child Health hospital in Vientiane found while all deliveries should be recorded into a hard-copy logbook, logbooks were not always available and date of birth often limited to year of birth [21]. Our research also affirms other studies which suggest there is often confusion among healthcare providers regarding the classification of stillbirth [4,6,7,21–23]. Another study in Lao PDR of five hospitals examined the causes and incidences of neonatal diseases and deaths also found inconsistency in documentation, a lack of differentiation between stillbirths with maceration and intrapartum stillbirths and the potential for some neonatal deaths to have been misclassified as stillbirth [23].

Increasing adequate investigation of stillbirth is a key recommendation of the 2016 Lancet Ending Preventable Stillbirth Series [20]. Healthcare providers in our study also recognised the benefit of autopsy examinations to help identify causes of stillbirth causes and assist preventive efforts. While recognising the healthcare system is already overstretched, the potential to implement the WHO guidelines for perinatal death audit could be explored [24]. There may also be opportunities for minimally or non-invasive autopsies including, for example, external examinations by skilled clinicians, ultrasound scan, post-mortem needle biopsy or laparoscopic autopsy which can provide valuable insights in the absence of complete diagnostic autopsies which are difficult to do in many LMICs [25]. More information on stillbirth would provide more accurate information for advocacy and the development of policies and programmes to reduce stillbirth and provide the necessary support for mothers, their families and healthcare professionals. The ability to provide explanations for stillbirth could also assist in reducing the stigma associated with stillbirth reported in this research [7,22,26].

The study made stark the lack of guidelines and training for health professionals in relation to bereavement care for mothers and their families, as well as inadequate emotional support for healthcare providers. Affirming other studies in LMICS [7], healthcare professionals recognised the need for appropriate bereavement care. Opportunities exist for both pre-and in-service education to improve the experience for women and their families as well as for healthcare professionals. Developing feasible, localised and culturally appropriate guidelines, informed by mothers’ perspectives, as well as providing pre- and in-service training on respectful maternity care [27] could also enhance the experience of women and healthcare providers. In the absence of formal guidelines, cultural and family beliefs and traditions are likely to determine practice and may prevent women from being offered the opportunity to hold, interact and bond with their stillborn baby, which can assist the healing process [28–30]. As in many other studies [31,32], healthcare professionals in Lao PDR highlighted the personal and professional impacts associated with this challenging area of practice. Intrapartum deaths, which are more common in LMICs, may add to these impacts [33]. Health system shortcomings, including under-resourced facilities, few opportunities for training and limited avenues for support exacerbate these impacts.

## Strengths and limitations

A strength of this study is the engagement with local Laotian healthcare partners and in-depth interviews with healthcare providers which generated valuable insights into the current state of stillbirth care in Lao PDR. Limitations of the study include confining participant recruitment and data collection to Vientiane Province and relying only on qualitative interviews. Other contexts are not addressed, including rural settings characterised by poverty and limited access to maternity care [9]. Another limitation is healthcare professionals were interviewed at their place of work which could have introduced some bias in responses although interviews took place in an area where confidentially could be assured. Also, our study focussed on healthcare providers and mothers’ perspectives is therefore missing.

## Conclusion

Efforts to prevent stillbirth are crucial, but so are high-quality bereavement care for women and adequate support for care providers after stillbirth. Shortcomings in relation to care and management following stillbirth exist in most countries, but challenges are exacerbated in LMICs due to scarce health system resources. Insights gained from healthcare providers in Lao PDR highlight a need for action to address current gaps relating to accurate recording of stillbirths, training and support for healthcare providers and guidelines for care of women and families.

## Supplementary Material

Supplemental Material

## Paper context

Globally, lower-middle-income countries carry the largest burden of stillbirth but most stillbirth research focuses on high-income settings. This study examined health professionals’ experiences of providing stillbirth care Lao PDR a lower-middle-income country. Limited health system resources and social stigma contribute to care that does not meet the needs of women following a stillborn baby. Training health professionals and community awareness raising may lead to more responsive care more positive experiences for healthcare providers.
