# Supplementary material for: Stillbirth in Lao PDR: a healthcare provider perspective
Source: Glob Health Action. 2020 Aug 3;13(Suppl 2):1786975. doi: 10.1080/16549716.2020.1786975 (PMC7480497; doi:10.1080/16549716.2020.1786975)
Supplement: Supplemental Material [file ZGHA_A_1786975_SM0697.docx]

**Supplementary material 1**

**Stillbirth in Laos: Perspectives of healthcare providers**

**Qualitative interview guide**

Being an exploratory study, the interviews will consist of a series of broad open-ended questions designed to elicit the views and experiences of the interviewee and to identify issues that they view as most relevant. The following topics will be covered but, in keeping with qualitative data collection techniques, there will be scope for flexibility.

1. Introduction and preamble [standard script to be used by interviewer]
2. Ensure participants about their privacy
3. Request for permission to audio-record interview (and reminder that the participant can stop the interview at any time)
4. Overall perspectives:

- What is stillbirth? How is stillbirth defined?
- How is stillbirth classified?
- In your experience, how common is stillbirth? For example, how many stillbirths occur in a year?
- What do you think are the main causes of stillbirth?
- What happens when a stillbirth occurs in your health care facility?
- What do you think are the impacts on the families?

1. Stillbirth review and investigation:

- How is stillbirth diagnosed in your healthcare facility?
- Are there any stillbirth reviews? if not, why? What are the barriers?

1. Bereavement care after stillbirth:

- What kind of support, do you think the parents need after stillbirth? (for example: emotional support, social or financial)
- What support do you or your facility offer to the parents? ( for example, education about family planning, advice on healthy pregnancy)

1. Stillbirth management and Impact on healthcare workers:

- How is stillbirth managed in your facility?
- How do you think stillbirth affect health care providers? (e.g. emotional impact and how does it impact their ability to care)
- What support or training opportunities exist in your facility, or might be helpful for staff in relation to stillbirth?
- Are there any guidelines for managing stillbirth in place or available?

1. Recommendations and Suggestion:

- Do you think healthcare workers need to be more supported? How?
- What areas do you think need improvement? (for example, more training for healthcare workers, ANCs)

1. Is there anything you would like to add?
